# 960 Statewide Landscape Analysis of Home Oxygen Therapy Related Fires and Burn Injuries

**DOI:** 10.1093/jbcr/iraf019.491

**Published:** 2025-04-01

**Authors:** Karla Klas, Mary Auber

**Affiliations:** University of Michigan, Michigan Medicine Trauma Burn Center; Saint Mary’s College

## Abstract

**Introduction:**

Studies show home oxygen therapy (HOT) related fires and injuries are increasing. However, observational data indicate these reports underestimate true incidence rates. This project aimed to analyze statewide HOT fire and burn incidents to provide a data tracking roadmap, identify gaps and inform risk reduction strategies.

**Methods:**

Liaisons of multiple statewide data sources were contacted to query HOT-related fires and injuries between 2020 - 2023: fire marshal via state-specific National Fire Incidence Reporting System (NFIRS); injury epidemiologist via death certificates, hospital admissions and ED visits; fire inspectors via real-time fatal fire reporting system app; prevention coordinators at 5 burn surge hospitals via registry or medical record review; and trauma system epidemiologist via EMS and trauma registries. Liaisons conducted manual searches of free text fields using 14 variations of HOT terms. State NFIRS queried “equipment involved in ignition – 416 oxygen administration equipment.” ICD-10-CM codes used for HOT injuries in registries were identified. Results were provided as deidentified aggregate data.

**Results:**

There are no specific ICD-10-CM HOT injury codes: Of 13 codes reported by registrars, the most used were X04 exposure to ignition of highly flammable material; X08/X09 exposure to unspecified smoke, fire, flames; and X00 exposure to uncontrolled fire in building. Terms in free text fields most used were “home oxygen” or “supplemental oxygen” often paired with “smoking.” Statewide NFIRS data revealed a mean of 3.5/year HOT fires (range 0-6), with a total of 10 injuries and 2 deaths. Fire inspectors reported mean 10.3 HOT deaths/year (range 5-15), representing 9.7% of residential fire fatalities and 10% of fatal residential fires. Death certificates were mean 10.3 HOT deaths/year (range 7-13) with 93.3% attributed to smoking. Trauma system data excluded burns, hence no HOT reported. Four hospitals (2 ABA burn verified) reported mean 17.9/year (range 13-31) each, with combined 4-year total of 285 (range 51-124) HOT admissions. This represents 33% of all HOT patients compared to published national burn center data. State epidemiologists and 1 hospital could not identify HOT injuries in hospital admissions, ED visits, and EMS data due to no specific coding. Large variances in data reporting, availability, and minimum number suppression requirements were noted.

**Conclusions:**

Statewide analysis revealed HOT deaths are occurring 20x more than reported in state NFIRS data. Burn surge hospital HOT admissions indicate published national burn center data greatly underestimate true HOT incidence rates. The lack of specific ICD-10-CM, optional use of fire incident codes, plus gaps in EMS and trauma data greatly impair tracking of HOT fires and injuries.

**Applicability of Research to Practice:**

Validates the need to establish injury codes, advocate for improved statewide reporting, and implement increased risk reduction strategies for HOT.

**Funding for the Study:**

N/A

